# Histories of Two Cases of Acute Inflammation of the Spinal Marrow

**Published:** 1821-04

**Authors:** 


### *Histories of two Cases of acute Inflammation of the Spinal Marrow.* By Dr. Pinel (the younger). Originally read to the Society of the College of Physicians of Paris.

MARY BRISSET, twenty-seven years of age, having always enjoyed good health, though of very acute nervous susceptibility, was accused of theft in the house in which she lived as a servant, and discharged in consequence of the false suspicions entertained respecting her. Her menses, which had flowed for three days, were immediately suppressed. She was deeply affected by the unmerited treatment she had received. On the third day from this time, she was found in her bed in a state of complete suspension of the functions of the sense and the understanding. She was then taken to the Hôtel-Dieu. After remaining there six weeks, the stupor disappeared; but a state of demency remained, and she was, consequently, transferred to the Salpetriere, August 18, 1818. At the time of her entry here, she was in the following state: Her look was bewildered; she had difficulty in articulating words; her answers were slow, difficult, and but rarely appropriate; there was great want of energy, but not paralysis, of all the limbs; she lay in a state of almost continual quietude, though she sometimes burst into violent fits of anger and impatience. The organic functions were executed perfectly and vigorously. But slight variations in the state of the patient were witnessed during fifteen months; it was only observed that she became very fat. On the 15th of January, 1820, she was seized, in the evening, suddenly, with convulsions. On the following morning, her mouth was found to be enveloped in froth; her eyes turned upwards; the jaws were spasmodically closed, except that the teeth were grated together; the trunk of the body agitated with convulsive shocks, repeated three or four times in a minute; the limbs were motionless, not at all participating in the convulsions of the body; the pulse full, frequent, irregular, and tumultuous; respiration short, oppressed, and precipitate; the alvine dejections involuntary; and the whole of the body covered by an abundant sweat, of a strong and tenacious odour, rising in vapour from the patient. The state of carus was profound. For three days, the convulsions of the trunk were continually repeated, appearing to increase in strength in the evening, and accompanied then by a febrile paroxysm. The other functions were similarly disordered. The patient died on the morning of the 18th of January, without having had any intermission of the symptoms above designated.

*Examination of the body, thirty-six hours after death.*

*The exterior.*—Habit, very lusty. The muscles of the face not contracted.

*The head.*—The cranium thick, and injected; the dura mater thin, almost diaphanous; the longitudinal sinus gorged with blood; the arachnoid presented, throughout the whole extent of the frontal and parietal regions, traces of inflammation of very ancient date; the pia mater was thickened by layers of coagulated lymph, and imbued with purulent serous fluid. The cerebrum and cerebellum, though carefully examined, presented nothing particular; the ventricles contained but little serum; the cerebral substance was firm and tenacious.

The spine presented nothing remarkable in respect to its membranes; but, after having divided the dura mater of the spinal marrow throughout its whole length, it was easy to discern a pultaceous disorganization of the medulla, commencing in the situation of the fourth cervical vertebra, and terminating about the fifth lumbar. Throughout the whole of this extent, the nervous pulp was reduced to a sort of yellowish, diffluent, and inodorous, porridge; towards the lumbar region, it resumed its ordinary consistence, and was bathed in a little reddish-coloured serum. The thoracic and abdominal viscera were perfectly healthy in their appearance; the interior of the stomach was of a light rose colour; the uterus was very small.

*Second Case.*—Felicia Lepoigny, of a sanguine temperament, having menstruated at eleven years of age, had always enjoyed good health until her fifteenth year: at this period, she was much terrified at the entry of the Russians into the village where she resided, and at being eagerly pursued by one of them, in consequence of which she was seized with a fit of epilepsy. Paroxysms of this affection were afterwards repeated, at first only after long intervals, but these gradually became shorter, and her intellectual faculties rapidly declined. She was brought to the Salpetriere in 1816, in a state of complete idiotism, accompanied with epilepsy, the paroxysms of which recurred every fourth or fifth day. During four years, hardly any change of her condition was observed. On the 7th and 8th of January, 1820, the fits were very frequent; but after this they returned to their ordinary type, until the 23d of January, when the convulsions succeeded each other with inconceivable rapidity. Her face was red and tumid; the muscles of the eye unequally and irregularly contracted; the trunk was almost incessantly agitated by convulsions, whilst the abdominal muscles were convulsively contracted in an undulatory manner; the limbs presented incoherent, but not convulsive, movements; sensibility, and the intellectual faculties, were totally abolished; the pulse was frequent, irregular, and low; respiration short and laborious. The convulsions and the other symptoms persisted, with the same degree of intensity, during the 24th: on the 25th, in the evening, the patient expired.

*Examination of the body, thirty hours after death.*

*The exterior.*—The muscles of the face convulsively, but *equally*, contracted; the countenance is red, the capillaries gorged with blood; the hymen perfect, and very apparent.

*The head.*—The cranium thick, injected, and very firm; the dura mater adherent to the skull about the right parietal fossa; the arachnoid healthy, but injected. The cerebral substance, of the ordinary aspect and consistence, presents nothing to be noticed, but a general injection of its blood-vessels, and a remarkably small capacity of the ventricles, which contain a little serum. The cerebellum is somewhat soft, though apparently healthy.

The spine, on its cavity being laid open in its whole extent, presents a very intense injection of its venous vessels; the medullary substance is the seat, about the dorsal region, of a species of disorganization similar to that found in the case of Mary Brisset: the pultaceous disorganization commences, superiorly, in the cervical region below the origin of the nervous plexus supplying the upper extremities, and it terminates inferiorly in the lumbar region; between these two points, the nervous mass is reduced to a fluid and yellowish porridge: in the rest of its extent, the spinal marrow is of the ordinary appearance.

*The thorax.*—The lungs are healthy; but the right one presents a cicatrix at about the middle of its costal surface.

The abdominal viscera presented nothing remarkable.

